# Longitudinal Variation in Population Structure, Growth, and Reproductive Characteristics of *Squalidus argentatus*, Driven by Cascade Dams in the Largest Tributary of Dongting Lake

**DOI:** 10.1002/ece3.74081

**Published:** 2026-07-27

**Authors:** Yanfei Huang, Yiyang Gao, Yang Zhengfei, Dongzhi Liu, LiLi Xie, Deliang Li

**Affiliations:** ^1^ College of Fisheries Hunan Agricultural University Changsha China

**Keywords:** age, Dongting Lake, fecundity, otolith, trade‐off

## Abstract

Cascade dams would affect directly and indirectly life history traits of fish through altering flow regime and habitats. Growth patterns and reproductive strategy of *Squalidus argentatus* were studied at the Yongzhou above cascade dams, Hengyang heavily regulated by cascade dams, Zhuzhou influenced by cascade dams and tributaries, and Changsha sections below cascade dams of the Xiangjiang River using otolith microstructure. Body size (range 46.9–146.9 mm) and age structure of 920 individuals showed an increasing trend downstream. The growth rate was significantly lower in the Hengyang section (0.099/year) than in the Yongzhou section (0.23/year). The absolute and relative fecundity were significantly higher in the Yongzhou section (6405 eggs and 64.5 eggs/mg) and Changsha (6156 eggs and 59.4 eggs/mg) than Hengyang (3092 eggs and 27.0 eggs/mg) and Zhuzhou (3378 eggs and 32.0 eggs/mg) sections. Egg size showed a significantly decreasing pattern from Yongzhou toward the lowest section. The results indicated that the population in Yongzhou showed good growth condition and strong reproductive potential; individuals in Hengyang had poor growth and adopted a strategy of low fecundity and large egg size; fish in Zhuzhou were in good growth condition but trade‐off chose large egg size; and individuals in Changsha showed low growth condition but selected producing a larger number of smaller eggs. We think that growth and reproduction of *Squalidus argentatus* have been influeced by cascade dams In Hengyang, Zhuhou, and Changsha. However, tributaries and a long distance between dams mitigated growth in Zhuzhou, and estuary habitats would be favorable for offspring survival and sustain the high fecundity of fish in the Changsha. The findings suggest that the construction of cascade dams should consider distance and location to minimize impacts.

## Graphical abstract

The Dongting Lake and the Xiangjiang River.

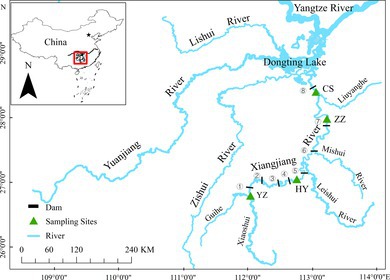

## 1 Introduction

The life history traits of fish include age structure, longevity, growth, size at maturity, spawning periods, fecundity, and egg size. These parameters are fundamental for understanding the status of a population, assessing its resilience to disturbance, and determining the life‐history strategies that are critical to developing sustainable fisheries management (Wakefield et al. 2015; Haarr et al. 2018; McNicholl et al. 2018). Life history traits vary in space and time and in response to divergent environmental conditions, including water temperature, food supply, fluctuations in flow rates, microhabitats, and population density (Tedesco et al. 2009; Nakajima and Onikura 2016; Adams et al. 2018; Tuor et al. 2020; Yang et al. 2026). The construction of large dams and small hydroelectric plants on rivers and tributaries worldwide has significantly altered aquatic habitats, ecological processes, physical attributes, flow regimes, and biota (Smith et al. 2017; Chen et al. 2023). Alterations in natural river flows and habitats, decreased water temperature, and reduced nutrient transport by dams, can affect the growth, reproduction, and survival of fish, both directly and indirectly (Korman and Campana 2009; Cheng et al. 2018). Studies have shown that dam construction can impact the population structure by altering flow regimes, leading to decreases or increases in growth (Jacquemin et al. 2015; Kelly et al. 2017; Tonkin et al. 2017; Whiterod et al. 2018). Body size distributions and age structure in regulated rivers differ from those in naturally flowing rivers, as well as in areas above and below dams (Enders et al. 2017; Huang et al. 2022). The effects of dams on age stucture, growth, and fecundity of freshwater fishes along longitudinal gradient are less well studied. A thorough understanding of variation in life history traits of freshwater fishes in a regulated river can be used to reveal the impacts of regional and small‐scale ecological processes on the population dynamics of species of concern, providing insights into conservation and management of fishery resources (Nakajima and Onikura 2016; Adams et al. 2018).

The Xiangjiang River is the largest tributary of Dongting Lake, the second largest lake in China. The lake is located in the middle reaches of the Yangtze River. The Xiangjiang River supports high fish diversity and abundant fishery resources due to high water discharge, with 147 fish species and approximately 40 commercial fishes. The plentiful lotic habitats in the middle and lower reaches once provided critical spawning habitat for river–lake migratory fishes, including the four major Chinese carps, *Parabramis pekinensis* , *Elopichthys bambusa* , and so on. The eggs and larvae from the Xiangjiang River drift into Dongting Lake and become an important supplemental source of fishery resources (Ding et al. 2010). Since 1999, eight cascade dams have been established in the Xiangjiang River, transforming the lotic habitats into slow‐flowing, lentic habitats. Water discharge and velocity have decreased in the reaches between cascade dams (Cao et al. 2019). Moreover, the operation of cascading dams results in hourly and diel variation in water discharge, velocity, and level, creating very unstable environmental conditions. As a result, species diversity has declined, and the fish community structure has been altered. Since 1970, migratory fish populations significantly declined, with marked decreases in egg and larval abundance (Ding et al. 2010). The altered environmental conditions suppress the growth and reproduction of fish inhabiting the regulated reaches of the Xiangjiang River. However, few studies have evaluated the growth and fecundity of fish or compared life history traits across different reaches concerning the effects of tributaries or the distances between cascade dams.

*Squalidus argentatus* is a small, widely distributed cyprinid fish species. In china, its distribution extends from the Heilongjiang River in the north to the Pearl River in the south (Wang et al. 2019; Liu et al. 2024). *Squalidus argentatus* is the dominant species in the middle and lower reaches of the Yangtze River and its tributaries. It can be found in a variety of habitats, but it prefers to inhabit slow‐flowing and lentic water bodies, spawn in lotic habitats, and produce drifting eggs (Wang et al. 2025). The fish is characterized by a short lifespan, early maturation, and low fecundity, traits that indicate strong adaptation to environments with frequent and intense disturbance. *Squalidus argentatus* is highly adapted to the Xiangjiang River environment and can be collected across habitats/reaches. *Squalidus argentatus* is a key ecological species within the Dongting Lake watershed, comprising a significant proportion among in the top 10 species across its four major tributaries. Investigations into fishery resources from 2023 to 2024 have revealed that *S. argentatus* comprises a significant component of annual catches, ranging from 15.0% to 35.3% in the mid‐stream and from 17.3% to 24.2% in the downstream areas of the Xiangjiang River (Gao et al. 2026). A 10‐year fishing ban, initiated in 2020, is currently in effect in the Yangtze River and its associated lakes and tributaries. And *Squalidus argentatus* in the Xiangjiang River has grown and spawned without fishing pressure for 5 years, and the current population structure has little relation to fishing intensity. Spatial patterns in the life history traits of *S. argentatus* would primarily reflect variation in environmental conditions resulting from dam construction.

Information on the life history traits of fish in the Xiangjiang River is limited, and the impacts of cascade dams urgently need to be assessed for fishery conservation and management. The aim of the present study was to examine the effect of cascade dams on longitudinal patterns in population structure, growth, fecundity, and egg size of *S. argentatus* along the Xiangjiang River. For this purpose, we first compared size and age structure, growth rate, fecundity, and egg size in four sections of the Xiangjiang with variable degrees of influence from dam constructions. According to the comparisons, the relationship between the patterns of population structure and growth and the flow regime regulated by cascade dams was discussed. Also, the question of how the trade‐off strategy between egg size and fecundity responded to habitat and environmental conditions in different reaches of the Xiangjiang River would be analyzed. The data would guide how to mitigate the effects of dam construction on fishery resources.

## 2 Materials and Methods

### 2.1 Study Area

The Xiangjiang River, with a length of 948 km and a drainage area of 94,660 km^2^, is the largest river in Hunan Province and is located in southern China and belongs to the middle Yangtze River floodplain ecosystem. The river originates in the southern part of Hunan Province and flows northward into Dongting Lake. The upper reaches (known as the Xiaoshui River) flow about 354 km and are confluent with a main tributary (the Guihe River) at Yongzhou; thereafter, the river is known as the Xiangjiang River. The middle reach is from Yongzhou until Hengyang (at Hengshan), with a length of 450 km. The lower reaches of the river extend from Hengyang to Changsha, covering approximately 290 km. Eight cascade dams were built between 2002 and 2016, with reservoir volumes of 0.85–6.75 hm^3^ and installed power of 5.2–14 MWh. These dams are run‐of‐river hydroelectric stations that conduct daily regulation of water discharge. The 10 main tributaries flow into the middle and lower reaches of the Xiangjiang River and have lengths ranging from 104 to 453 km. A large number of small hydropower plants operate in these low‐ to medium‐order rivers. The Xiangjiang River lies in the subtropical monsoon climate zone and experiences seasonal changes. The annual mean discharge rate is 2300 m^3^/s, and 70%–75% of the total runoff occurs during the rainy season (during April and September). The water temperature ranged from 0.17°C to 39.94°C, with a mean ± SD of 21.48°C ± 8.80°C (measured daily from September 2024 to September 2025 at Changsha).

**FIGURE 1:**
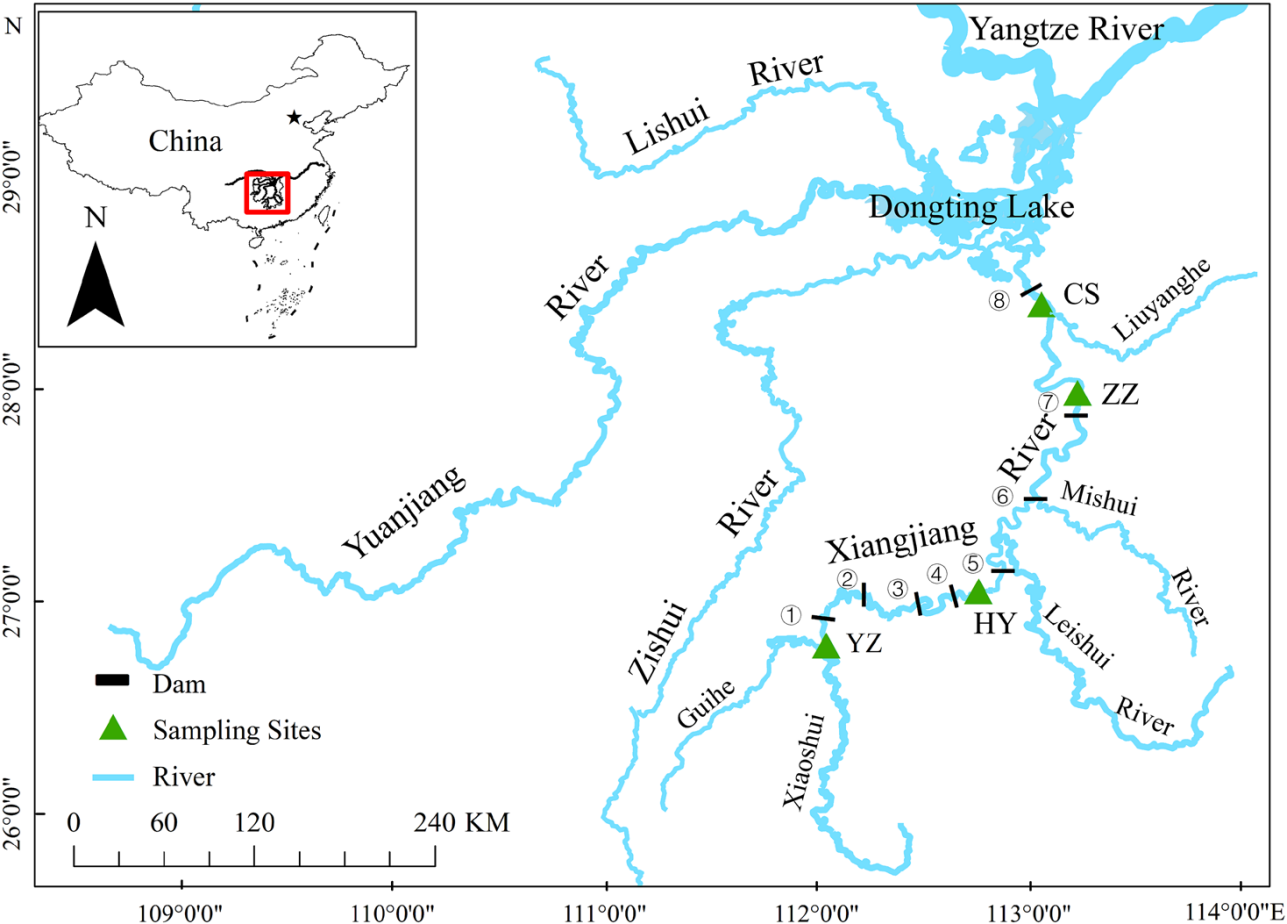
Map showed the four sampling sites (YZ, Yongzhou; HY, Hengyang; ZZ, Zhuzhou; CS, Changsha) and eight hydropower stations ((1), the Xiaoxiang Station; (2), the Wuxi Station; (3), the Xiangqi Station; (4), the Jinweizhou Station; (5), the Tugutang Station; (6), the Daduyuan Station; (7), the Zhuzhou Station; (8), the Changsha Station) in the middle and lower reaches of Xiangjiang River, a tributary of Dongting Lake. Red rectangle indicates the location of Dongting Lake and its tributary.

The collections were carried out at four sampling sites, including one “least‐impacted” site at Yongzhou above dams and three sites located between cascade dams along the middle and lower reaches of the Xiangjiang River (Figure 1). The most upstream sampling site at Yongzhou (26°32′ N, 112°37′ E) is located at the confluence of the Xiaoshui and Guihe Rivers, approximately 22.56 km above the uppermost dam (Xiaoxiang). The second sampling site at Hengyang (26°33′ N, 112°27′ E) is located 14.68 km downstream of the fourth dam (Jinweizhou) and 35.26 km upstream of the fifth dam (Tugutang). The distance between Yongzhou and Hengyang is 195.06 km, with four cascade dams, each about 50 km apart. Two main tributaries with a length of 118 and 302 km flow into the river at this point. The third sampling site at Zhuzhou (27°39′ N, 113°06′ E) is located downstream of the seventh dam (Zhuzhou), which is 101 km downstream of the sixth dam and 135 km above the last dam. The mainstream between Hengyang and Zhuzhou is approximately 225 km long, and it receives three main tributaries with lengths of 453, 194, and 296 km, respectively. Five major tributaries with lengths of 168, 198, 103, 230, 181, and 144 km respectively flow below the Zhuzhou dam. The lowermost sampling site at Changsha (28°19′ N, 112°54′ E) is immediately downstream of the last Changsha Hydroelectric Station and at a distance of 15 km from the estuary at Qiaokou from which it flows into the Dongting Lake.

Daily water discharge was recorded by the Hunan Hydrology Station (http://yzt.hnswkcj.com:9090/#/) at Yongzhou, Qiyang close to Hengyang, Zhuzhou, and Changsha. Water discharge was relatively high in April and August and very low (near zero velocity) during September and March at Yongzhou, Hengyang, and Zhuzhou. Water discharge was higher at Zhuzhou than at the Yongzhou and Qiyang sections, but it had the same pattern and magnitude at Yongzhou and Qiyang. No water discharge was observed at Changsha Hydrology Station, where the discharge was measured in the impounding reservoir. Water discharge at Yongzhou, Qiyang, and Zhuzhou showed daily variation due to the regulation of cascade dams, with abrupt decreases or increases during the low‐water season, but was altered by precipitation and showed natural patterns during the high‐water season (Figure A.1). The water velocity is relatively high at Yongzhou, Zhuzhou, and Changsha and low at the Hengyang section. The densities of phytoplankton and zooplankton showed an increasing downstream trend and were higher in the reservoir area than below the dams at Zhuzhou and Changsha. The widths of the sampling sections increased in the downstream direction (Table A.1).

### 2.2 Sampling and Laboratory Processing

A 10‐year fishing ban is in effect in Dongting Lake and its four main tributaries including the Xiangjiang River, Yuangjiang River, Zishui River, and Lishui River. To timely understand fishing ban effectiveness, fishery resource monitoring investigations are allowed to be conducted twice a year, so fish specimens were caught during the spawning period and the feeding period of most species in the Xiangjiang River. *Squalidus argentatus* was collected in May and October 2023 and May 2024 at Yongzhou; in May and October 2023 and May and June 2024 at Hengyang; in May and September 2023 and May 2024 at Zhuzhou; and in June and October 2023 and May 2024 at Changsha. Most specimens of variable size were captured by set gillnets (50 m length × 1.2 m width, 2‐cm mesh), with a few individuals from the catches of benthic fyke nets (18 m length × 0.33 m width × 0.45 m height, 0.8‐cm mesh). All specimens were initially preserved in ice and then frozen until dissection. Each fish was measured for standard length (SL, 0.1 mm) and body weight (BW, 0.01 g). The specimens were dissected, and the gonads were evaluated macroscopically to determine sex and maturity stage (II, ovary was small, no granular eggs were seen; III, ovary became large, oocytes were visible as granular; IV, ovary was yellow, oocytes were big; VI, oocytes were transparent and dissociative). Gonad mass (0.01 g) and gutted gonad mass (0.01 g) were measured for mature females (phases IV and V). Sub‐samples (0.11–0.74 g) were taken from the anterior, middle, and posterior sections of the right ovary and preserved in a 5% buffered formalin solution for fecundity analysis. Both lapillus otoliths for each measured fish were extracted, cleaned of adherent tissues, and stored dry in labeled Eppendorf tubes for age determination.

In the laboratory, oocytes from preserved gonads were photographed under a dissecting microscope (Olympus SZX16) connected to a PC with a digital camera, and the diameters of mature oocytes were measured using a measurement analysis system in the Image‐Pro Plus program 6.0. The mature oocytes were counted using Carl Zeiss Vision (AxioVision 3.1). The gonadosomatic index (GSI), a measure of reproductive maturity, was calculated as the gonad weight multiplied by 100 and divided by the body weight.

Absolute fecundity (AF) was assessed by estimating the total number of oocytes for each matured female using the following equation:

(1) $$\mathrm{AF}=\left(\text{number of mature oocytes in the subsample}\times \text{gonad weight}\right)/\text{the weight of the subsample}$$

Relative fecundity (RF) was estimated as the number of mature oocytes per body weight unit (g) and per body length unit (mm) by the following equation:

(2) RF=AF/body weight andAF/SL

About 50% of fish for each sampling section were selected for age determination. One otolith from each selected fish was mounted on a glass slide using transparent enamel resin. Both sides of the otolith were ground using wet carbide sandpaper and then polished with diamond sandpaper until the otolith core and growth increments became visible.

The otolith microstructure was examined using an optical microscope with reflected light against a white background. Otolith images were captured using the LCmicro Image Analysis software. Increment readings and measurements of otolith radius and increment widths were conducted along the posterior axis parallel to the anterior–posterior axis. A black primordium emerged on the anterior and ventral side of the lapillus, surrounded by a black area. The otolith core was composed of the primordium and the black area and is similar to that of *Hemiculter leucisculus* (Huang et al. 2022). The translucent and opaque bands formed alternately outside the otolith core. The first annual increment was demarcated by the initial opaque band, and the number of annuli corresponded to the count of opaque bands. Specimens captured in May and June exhibited opaque otolith margins, while those collected in September and October exhibited translucent otolith edges. The reproductive period extended from May to August, with the average hatching date estimated to occur in June. The age of fish with opaque otolith margins matched the number of annuli, and individuals with translucent otolith edges were aged as follows:

(3) $$\mathrm{Age}=\text{annuli}+\left(\text{capture month}-6\right)/12$$

The remaining fish were not aged by otolith microstructure analysis and therefore were estimated by an age‐length key (ALK), which used SL‐at‐age data and the number of fish in 10‐mm intervals (Huang et al. 2022).

### 2.3 Data Analysis

Body length frequency distributions were compared among four sampling sections using a Kruskal‐Wallis test, and each pairwise comparison was further analyzed with Post Hoc Comparisons when differences were significant. Body weight and length relationship was fitted to a power model:

(4) $$\mathrm{BW}=a{\mathrm{SL}}^{b}$$

where *a* and *b* were parameters. The analysis of covariance ANCOVA was used to test the heterogeneity of slopes of the SL‐BW relationships among populations with log‐transformed data and differences between intercepts of the regressions. Each pairwise was further compared, and the significance level was adjusted to *p* = 0.05/6, where 6 is the number of comparisons made, based on the Bonferroni correction. The condition was assessed based on the condition factor (*K*), which was calculated as:

(5) $$K=\left(\mathrm{BW}/{\mathrm{SL}}^{3}\right)\times 100$$

where BW is in grams and SL is in centimeters. One‐way analysis of variance (ANOVA) was used to compare condition values among sampling sections when data met normality. Each pairwise comparison was further conducted using Tamhane's T2 test when the variance could not reach homogeneity.

The Kruskal‐Wallis test was used to analyze differences in AF and RF across populations in the sampling sections, as those AF and RF data were not normally distributed. Pairwise comparisons were performed between sections. Differences in oocyte diameter (OD) were analyzed by one‐way ANOVA, and each pairwise difference was further compared using Tamhane's T2 test. Regression analysis was used to determine the relationship between various body descriptors, i.e., between SL and AF, RF, and OD, and between BW and AF, RF, and OD.

The von Bertalanffy growth (VBGF) function was applied to the SL‐at‐age data at the four sampling sites. The VBGR was fitted by the equation:

(6) $$L_{t}=L_{ꝏ}\left[1-\exp^{-k\left(t-t0\right)}\right]$$

where *L*_*t*_ is the SL at the age time *t* (age), *L*_∞_ is the theoretical asymptotic length, *k* is the growth coefficient (year^−1^), *t*_0_ is the theoretical time at 0 length.

The parameters of the equation were estimated by least squares, and residual sum of squares (RSS) was used to compare the VBGF value among the sampling sites. The VBGF values were further compared between each pair of sampling sites using the Bonferroni correction, with the significance level adjusted to *p* = 0.05/6. Furthermore, a growth performance indice *Φ* = log_10_ *k* + 2 log_10_ *L*_∞_ was calculated as an indice of growth performance.

## 3 Results

### 3.1 Age and Growth

**FIGURE 2:**
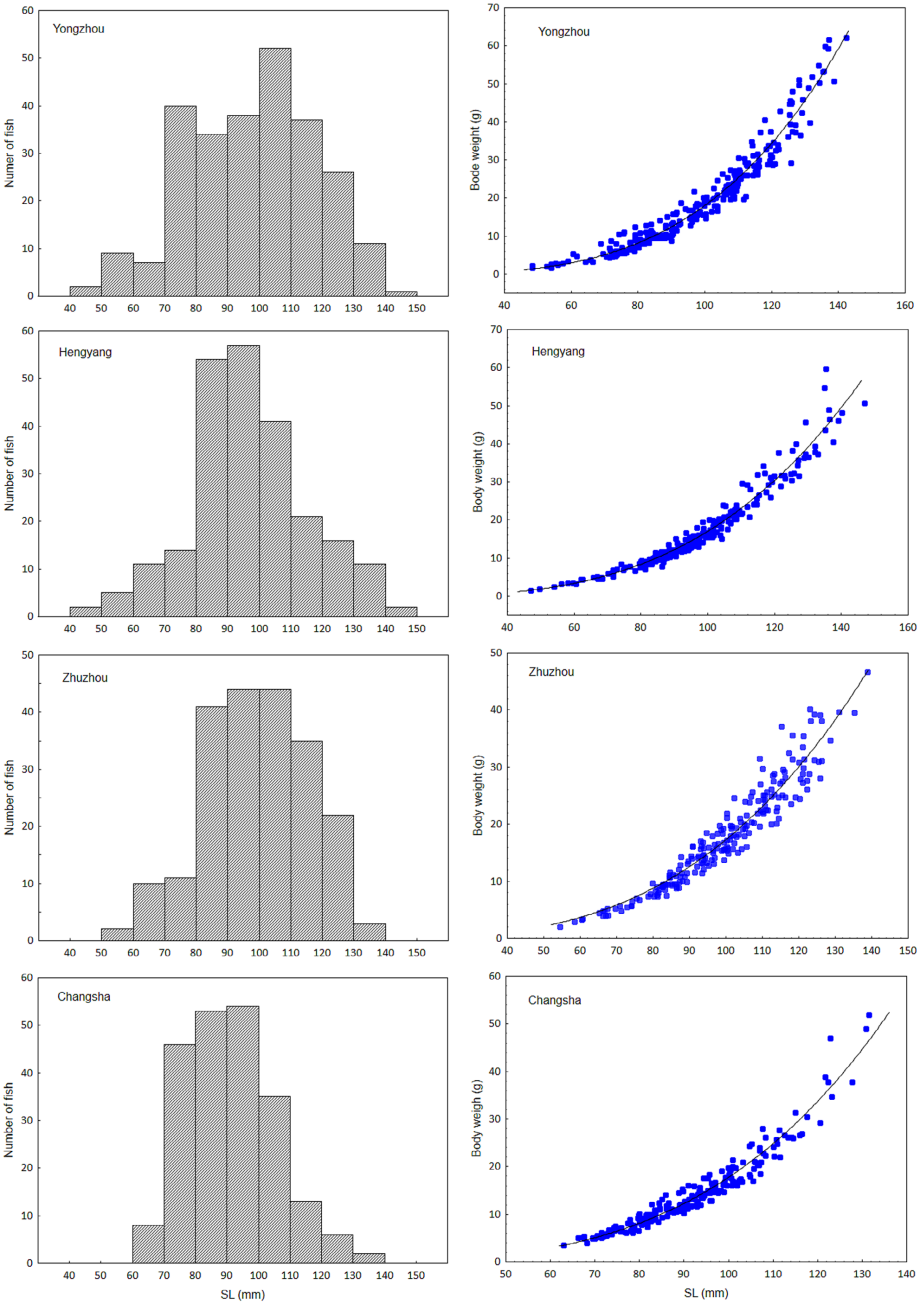
Size‐frequency distributions and the relationships between standard length (SL, mm) and body weight (g) of *Squalidus argentatus* collected at Yongzhou, Hengyang, Zhuzhou, and Changsha in the middle and lower reaches of Xiangjiang River.

A total of 920 individuals were collected from the four sampling sites in the Xiangjiang River (Table 1). The SL frequency distributions exhibited peaks in the ranges of 100–110 mm at Yongzhou, 80–100 mm at Hengyang and Changsha, and 90–110 mm size at Zhuzhou (Figure 2). The SL frequency distributions differed significantly among the four sampling sites (*H* = 32.04, *p* < 0.001), with the mean SL being significantly smaller at Changsha than at the other three sites (Table 1, *p* < 0.05). BW‐SL relationships of *S. argentatus* demonstrated positive allometric growth in the four sampling sites, and the corresponding equations were as follows (Figure 2):

$$\text{Yongzhou},\mathrm{BW}=1.543\times 10^{-6}{\mathrm{SL}}^{3.534}\;\left(F_{\left(2,255\right)}=9592.68,r^{2}=0.957\right)$$

$$\text{Hengyang},\mathrm{BW}=7.752\times 10^{-6}{\mathrm{SL}}^{3.171}\;\left(F_{\left(2,232\right)}=9323.76,r^{2}=0.956\right).$$

$$\text{Zhuzhou},\mathrm{BW}=15.08\times 10^{-6}{\mathrm{SL}}^{3.03}\;\left(F_{\left(2,210\right)}=6222.23,r^{2}=0.916\right)$$

$$\text{Changsha},\mathrm{BW}=1.674\times 10^{-6}{\mathrm{SL}}^{3.513}\;\left(F_{\left(2,215\right)}=8557.27,r^{2}=0.949\right)$$

**TABLE 1:** A review of number, mean value (±SD), and range of standard length (SL, mm), body weight (BW, g), the condition factor (K) for all specimens, otolith radius (OR, μm) for aged fish, gonadosomatic index (GSI), absolute fecundity (AF, eggs), relative fecundity (RF, eggs/mm and eggs/g) of mature females, and oocyte diameter (OD, mm) of *Squalidus argentatus* measured of OD collected at Yongzhou, Hengyang, Zhuzhou, and Changsha in the middle and lower reaches of Xiangjiang River in 2023 and 2024.

		Yongzhou	Hengyang	Zhuzhou	Changsha
Number	920	257	234	212	217
SL (mm)	Mean ± SD	98.1 ± 20.4	96.8 ± 18.7	98.8 ± 16.4	91.0 ± 14.0
	Range	48.3–142.2	46.9–146.9	54.3–138.8	62.9–131.5
BW (g)	Mean ± SD	20.16 ± 13.31	17.32 ± 10.79	18.05 ± 9.00	14.20 ± 8.08
	Range	1.67–62.10	1.47–59.70	2.07–46.71	3.51–51.85
CF	Mean ± SD	1.81 ± 0.31	1.69 ± 0.17	1.71 ± 0.23	1.71 ± 0.21
	Range	1.13–2.56	1.21–2.41	1.28–2.43	1.24–2.54
Number	465	132	107	104	122
OR (μm)	Mean ± SD	934.0 ± 179.	891.1 ± 156.6	871.8 ± 129.3	851.6 ± 116.7
	Range	453.4–1312.9	515.3–1257.4	494.0–1159.0	628.8–1158.6
Number	290	93	61	46	90
GSI	Mean ± SD	16.57 ± 11.59	8.02 ± 2.46	9.12 ± 3.29	16.81 ± 7.29
	Range	5.74–51.0	5.31–16.10	5.46–22.45	6.3–38.23
AF (eggs)	Mean ± SD	6405 ± 5511	3092 ± 2811	3378 ± 1858	6156 ± 7054
	Range	1049–32,436	1013–12,246	1391–11,629	1016–31,253
RF (eggs/mm)	Mean ± SD	56.4 ± 41.2	27.0 ± 19.9	32.0 ± 16.5	59.4 ± 60.4
	Range	12.2–249.6	10.9–94.8	13.4–105.7	6.9–240.1
RF (eggs/g)	Mean ± SD	229.0 ± 130.5	128.7 ± 48.4	155.1 ± 64.3	304.1 ± 239.5
	Range	51.6–700.0	57.5–289.1	66.2–390.4	63.2–1034.9
Number	237	44	60	45	88
OD (mm)	Mean ± SD	0.92 ± 0.065	0.89 ± 0.069	0.84 ± 0.055	0.78 ± 0.089
	Range	0.73–1.04	0.68–1.05	0.75–0.96	0.64–0.96

These relationships were significantly different among sampling sites (Table 2). A pairwise comparison revealed a significant difference between Yongzhou and Hengyang (*p* < 0.05/6) and between Hengyang and Changsha (*p* < 0.05/6).

**TABLE 2:** The comparison of length‐weight relationships among the four sampling sections using an analysis of covariance (ANCOVA) after the data was log‐transformed.

Factors	df	MS	*F*	*p*
SL (covariate)	1	317.00	20346.85	< 0.001
Sampling site	3	0.13	7.88	< 0.05
Intercept	1	213.52	13704.76	< 0.001
Site × age (slope)	3	0.14	8.65	< 0.001

The condition coefficients differed significantly among the four populations (*F*_(3,916)_ = 13.35, *p* < 0.001, Table 1), with the value being significantly higher for fish at Yongzhou than at the other three sampling sites (*p* < 0.05).

**FIGURE 3:**
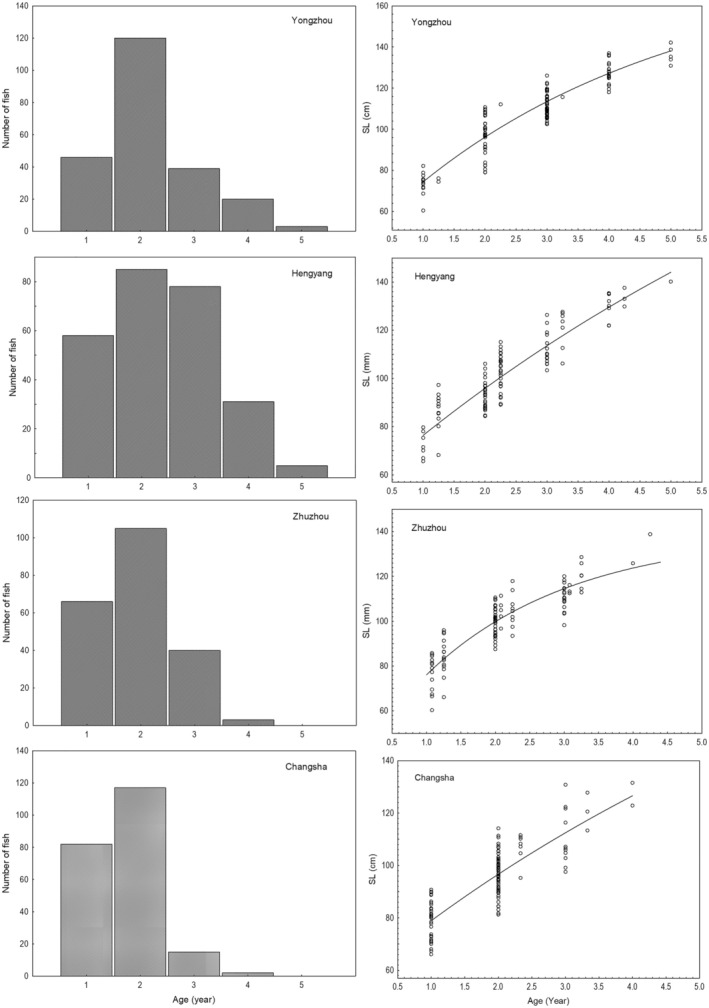
Age compositions, age‐at‐length plot and the von Bertalanffy growth curves fitted to the relationships for *Squalidus argentatus* collected at Yongzhou, Hengyang, Zhuzhou, and Changsha in the middle and lower reaches of Xiangjiang River.

*Squalidus argentatus* ranged in age from 1 to 5 years at Yongzhou and Hengyang and from 1 to 4 years at Zhuzhou and Changsha. The age compositions were dominated by the two‐year age class at Yongzhou, the two‐ and 3‐year age classes at Hengyang, and the two‐ and one‐year age classes at Zhuzhou and Changsha (Figure 3).

Otolith radius showed a positive linear relationship with SL (Table 1). The SL‐OR relationships were calculated as follows:

$$\text{Yongzhou},\mathrm{SL}=7.792+0.10\text{4OR}\;\left(F_{\left(1,132\right)}=953.4,r^{2}=0.88\right)$$

$$\text{Hengyang},\mathrm{SL}=16.037+0.09\text{7OR}\;\left(F_{\left(1,107\right)}=476.73,r^{2}=0.82\right)$$

$$\text{Zhuzhou},\mathrm{SL}=9.129+0.10\text{4OR}\;\left(F_{\left(1,103\right)}=461.11,r^{2}=0.82\right)$$

$$\text{Changsha},\mathrm{SL}=8.562+0.10\text{1OR}\;\left(F_{\left(1,122\right)}=360.48,r^{2}=0.75\right)$$

The intercept was statistically significantly different among the four populations (*F*_(3,7)_ = 4.732, *p* < 0.05), whereas the slope was not significantly different among populations (*F*_(3,7)_ = 0.77, *p* > 0.05).

A total of 465 fish aged from 1 to 5 years were used to estimate growth rates. The VBGF model was fitted to the SL‐at‐age relationship as follows: (Figure 3)

$$\text{Yongzhou},\mathrm{SL}=180.32\left(1-e^{-0.23\left(\mathrm{age}+1.314\right)}\right)\;\left(F_{\left(3,125\right)}=11097.50,r^{2}=0.86\right)$$

$$\text{Hengyang},\mathrm{SL}=283.27\left(1-e^{-0.099\left(\mathrm{age}+2.175\right)}\right)\;\left(F_{\left(3,104\right)}=7480.47,r^{2}=0.82\right)$$

$$\text{Zhuzhou},\mathrm{SL}=138.88\left(1-e^{-0.473\left(\mathrm{age}+0.68\right)}\right)\;\left(F_{\left(3,101\right)}=7008.20,r^{2}=0.78\right)$$

$$\text{Changsha},\mathrm{SL}=228.63\left(1-e^{-0.125\left(\mathrm{age}+2.439\right)}\right)\;\left(F_{\left(3,119\right)}=6050.3,r^{2}=0.68\right)$$

Growth curves differed significantly among the four populations (ARSS, *F*_(9,453)_ = 17.53, *p* < 0.001). The fish grew more rapidly at Zhuzhou than at Hengyang (*F*_(3,205)_ = 9.04, *p* < 0.05/6) and at Changsha (*F*_(3,220)_ = 6.20, *p* < 0.05/6), and there were no significant differences in growth between the other two pairs (*p* > 0.05/6). The growth performance indices were 3.87, 3.90, 3.96, and 3.83 for populations at Yongzhou, Hengyang, Zhuzhou, and Changsha, respectively.

### 3.2 Fecundity and Egg Size

Of the 920 collected individuals, 290 were mature females, ranging in age from 1 to 5 years. The standard lengths of females ranged from 60.5 to 142.2 mm, and their weights ranged from 5.3 to 62.1 g. The mean GSI was higher at Yongzhou and Changsha compared to that at Hengyang and Zhuzhou (Table 1). At Yongzhou, there was a greater proportion (50.5%) of mature females in the three‐year‐old age class; at Hengyang, in the two‐year‐old (44.3%) and three‐year‐old (32.8%) age classes; at Zhuzhou, in the two‐year‐old (58.7%) and three‐year‐old (39.1%) age classes; and at Changsha, in the two‐year‐old age class (74.4%).

**FIGURE 4:**
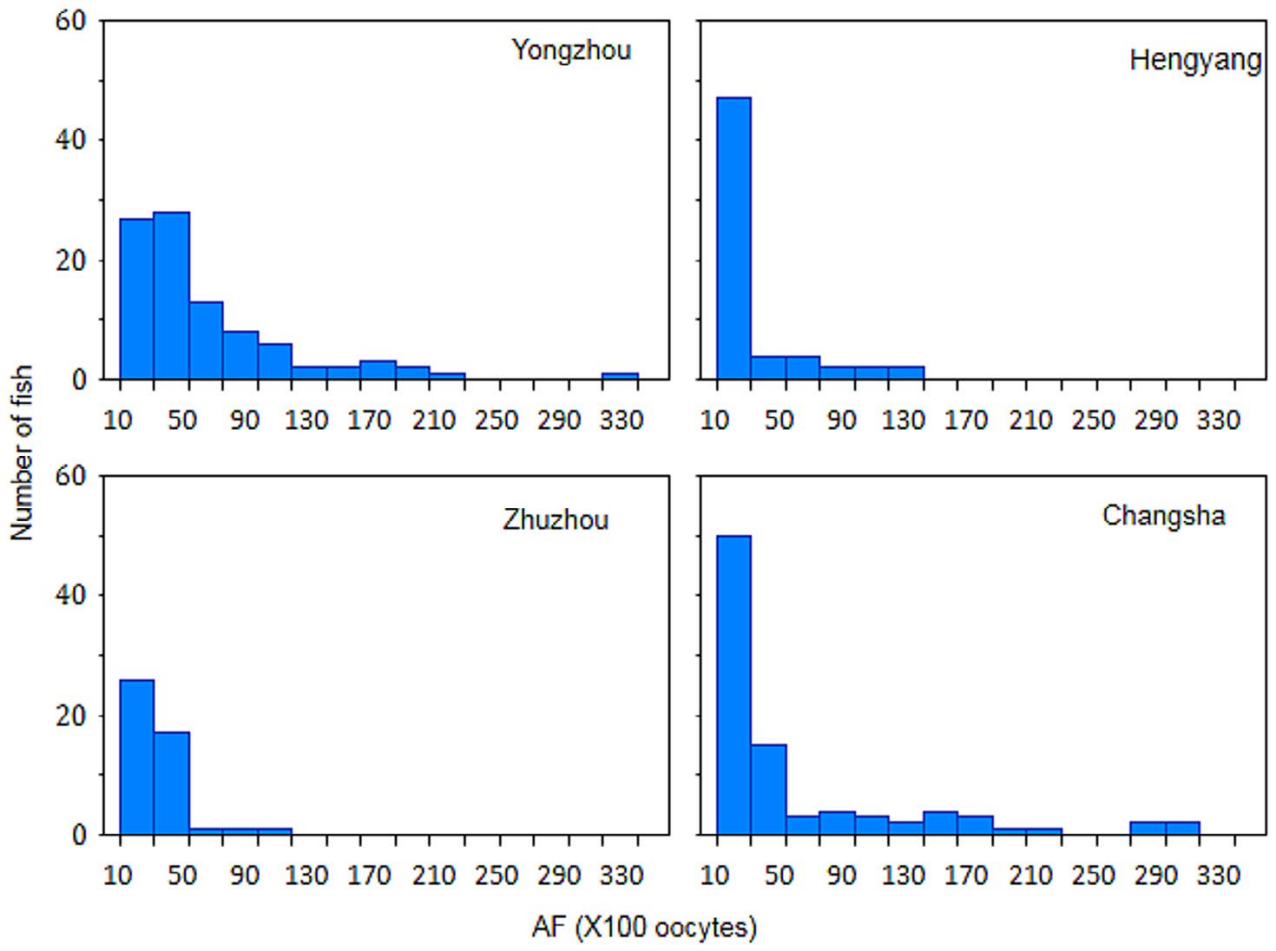
Frequency distributions of absolute fecundity (AF, oocyte) for mature females collected at Yongzhou, Hengyang, Zhuzhou, and Changsha in the middle and lower reaches of Xiangjiang River.

**FIGURE 5:**
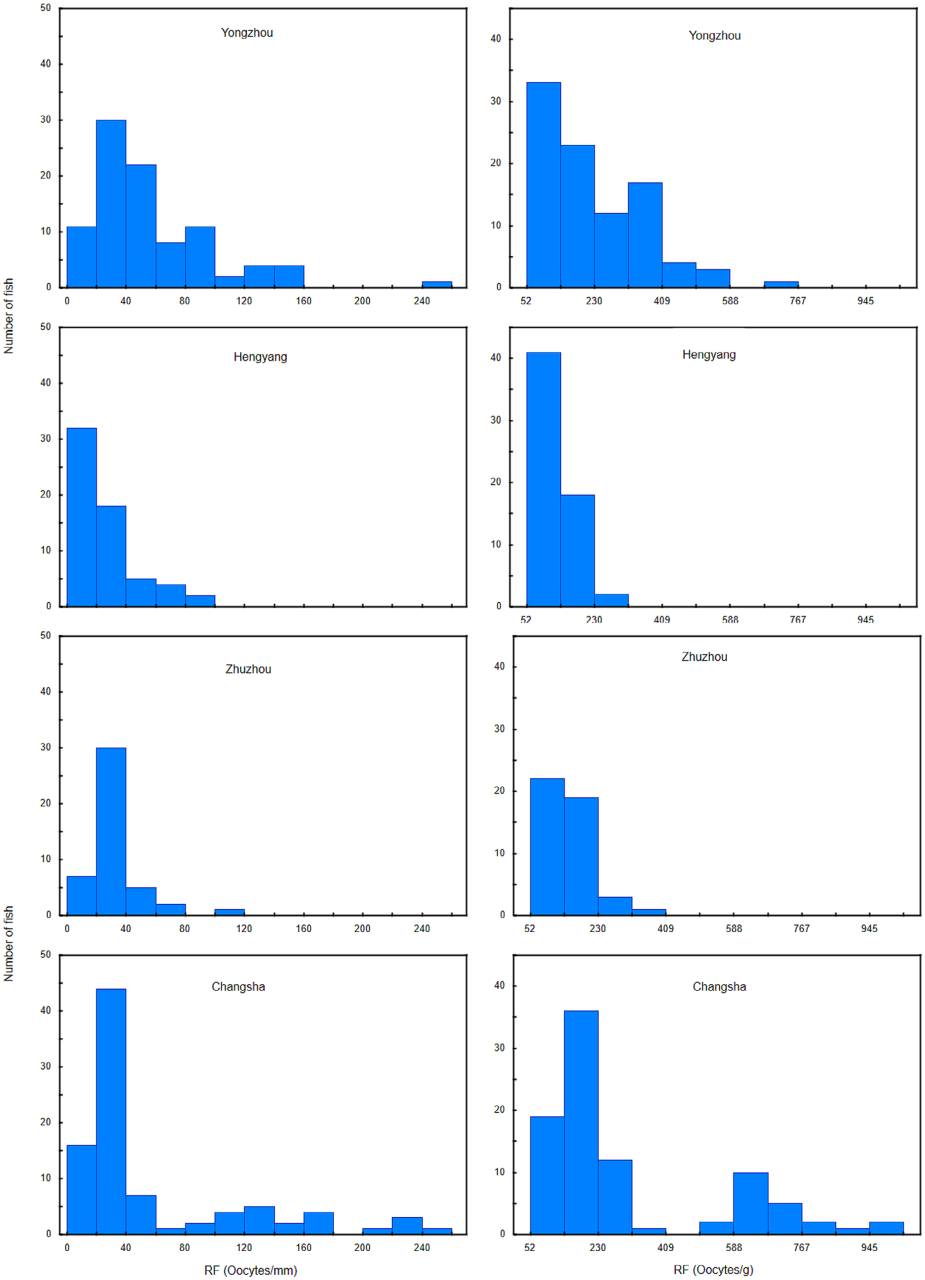
Frequency distributions of relative fecundity for standard length (RF, oocyte/mm) and for body weight (RF, oocyte/g) of mature females collected at Yongzhou, Hengyang, Zhuzhou, and Changsha in the middle and lower reaches of Xiangjiang River.

The absolute fecundity ranged from 1013 to 32,436 oocytes, with Yongzhou having the highest mean value (Table 1). AF frequency distributions showed a peak in the 3000–5000 oocytes group at Yongzhou and in the 1000–3000 oocytes group at the other three sites (Figure 4). Significant differences in AF were observed among the four sampling sites (*F*_(3,286)_ = 7.80, *p* < 0.001), and the AF of fish was significantly higher at Yongzhou and Changsha than at Hengyang and Zhuzhou (*p* < 0.05). The mean relative fecundity per mm SL and gram body weight were the highest at Changsha (Table 1). The frequency distributions of RF per mm SL showed dominant classes of 20–40 and 40–60 oocytes/mm at Yongzhou, 0–20 oocytes/mm at Hengyang, and 20–40 oocytes/mm at Zhuzhou and Changsha (Figure 5). There was a significant difference in RF for SL among the four sampling sites (*H*_(3,290)_ = 46.93, *p* < 0.001). RF per SL was significantly higher for fish at Yongzhou and Changsha than at Hengyang (*p* < 0.001) and significantly higher for fish at Yongzhou than at Zhuzhou (*p* < 0.05). The dominant RF class per gram of body weight was 50–150 oocytes/g at Yongzhou, Hengyang, and Zhuzhou and 150–250 oocytes/g at Changsha. There was a significant difference in RF for body weight among the four sampling sites (*H*_(3,290)_ = 52.59, *p* < 0.001), with significantly higher RF for body weight at Yongzhou and Changsha than at Hengyang and Zhuzhou (*p* < 0.05).

**FIGURE 6:**
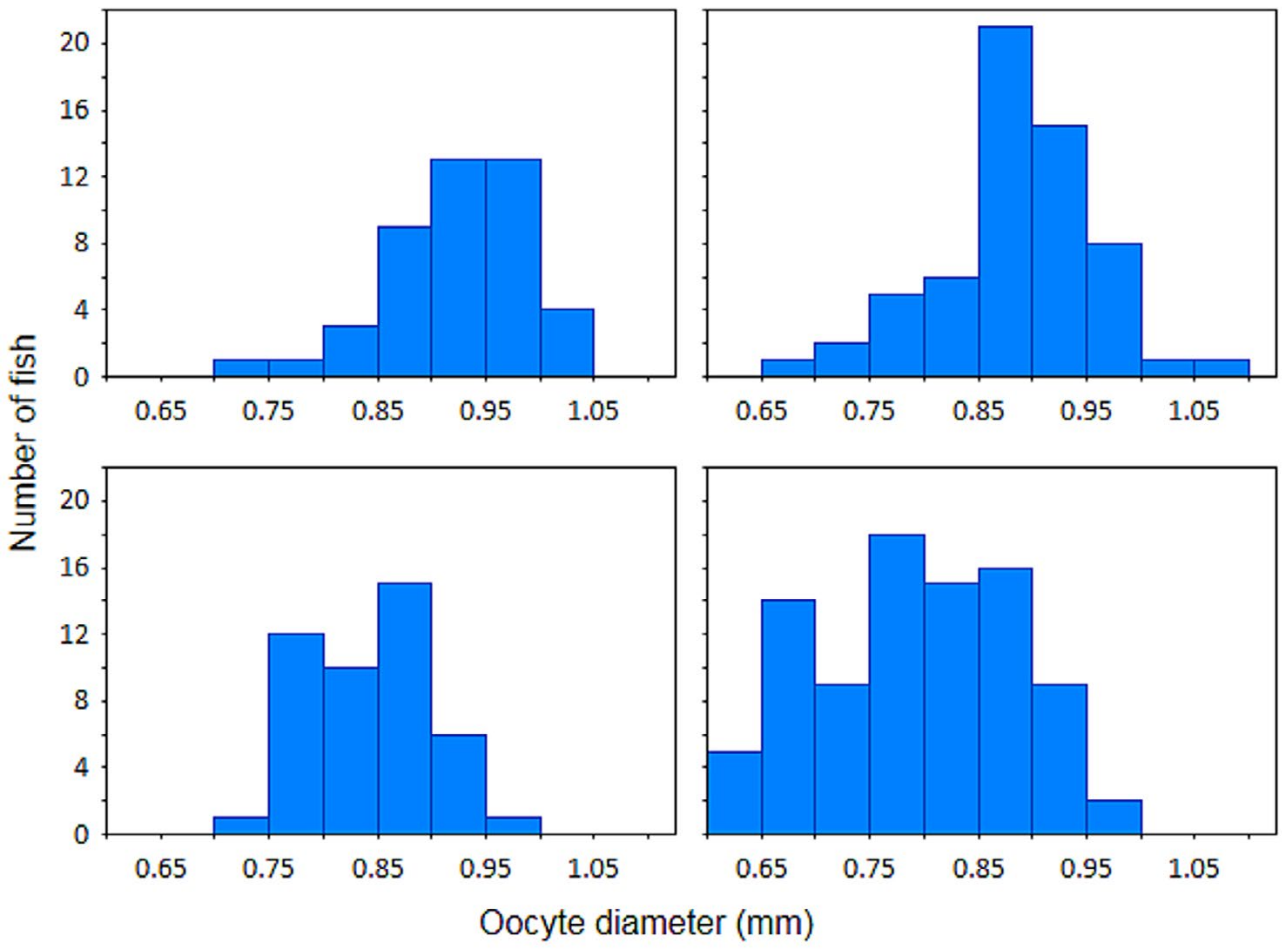
Frequency distributions of individual mean oocyte diameter (mm) of mature females collected at Yongzhou, Hengyang, Zhuzhou, and Changsha in the middle and lower reaches of Xiangjiang River.

Among 237 fish measured for OD ranged from 0.51 to 1.228 mm (mean, 0.841 ± 0.039 mm, *n* = 26,422). The mean OD exhibited a decreasing trend in the downstream direction (Table 1). Individual mean OD had a dominant class of 0.90–1.00 mm at Yongzhou, and the corresponding measurements were 0.85–0.95 mm at Hengyang, 0.85–0.90 mm at Zhuzhou, and 0.75–0.90 mm at Changsha (Figure 6). There was a significant difference in OD among the four sampling sites (*H*_(3,237)_ = 77.51, *p* < 0.001), with significantly higher mean OD at Yongzhou and Hengyang than at Zhuzhou and Changsha (*p* < 0.05).

Absolute fecundity was positively correlated with both SL and BW. Linear regressions were fitted to the AF‐SL and AF‐BW data, revealing variable coefficients of determination across the four populations (Table 3). At three sampling sites (excluding Yongzhou), OD showed no significant correlation with SL; however, it was positively correlated with BW, albeit with a low coefficient of determination. For the four populations, relative fecundity (oocytes/g) is significantly related to SL and BW, except for the RF‐SL relationship in Zhuzhou.

**TABLE 3:** The relationship between absolute fecundity (AF), oocyte diameter (OD), and relative fecundity (RF, oocytes/g) and body size (SL), body weight (BW) at Yongzhou, Hengyang, Zhuzhou, and Changsha in the middle and lower reaches of Xiangjiang River in 2023 and 2024.

Sites	AF‐SL relationship	*r* ^2^	OD‐SL relationship	*r* ^2^	RF‐SL relationship	*r* ^2^
	Function		Function		Function	
Yongzhou	SL = 97.69 + 0.002AF	0.29	SL = 149.64ED−31.62	0.21	SL = 98.78 + 0.17RF	0.17
Hengyang	SL = 92.31 + 0.004AF	0.61	−(*p* > 0.05)	—	SL = 90.26 + 0.59RF	0.52
Zhuzhou	SL = 99.99 + 0.001AF	0.09	−(*p* > 0.05)	—	−(*p* > 0.05)	—
Changsha	SL = 99.01 + 0.001AF	0.35	−(*p* > 0.05)	—	SL = 92.27 + 0.09RF	0.26

	AF‐BW relationship	*r* ^2^	OD‐BW relationship	*r* ^2^	RF‐BW relationship	*r* ^2^
Yongzhou	BW = 17.04 + 0.002AF	0.53	BW = 123.12ED−86.02	0.26	BW = 16.99 + 0.19RF	0.38
Hengyang	BW = 10.28 + 0.004AF	0.77	BW = 56.72ED−5.06	0.1	BW = 8.25 + 0.52RF	0.69
Zhuzhou	BW = 16.91 + 0.001AF	0.23	BW = 31.87ED−28.02	0.09	BW = 17.94 + 0.12RF	0.16
Changsha	BW = 12.23 + 0.001AF	0.62	BW = 36.34ED−10.69	0.15	BW = 12.24 + 0.10RF	0.49

## 4 Discussion

Notable differences in the life history traits of an important eurytopic fish were observed along the middle and lower reaches of the Xiangjiang River in this study. The distances between pairs of sampling sections were only approximately 100–200 km, but populations from the four sampling sections were isolated from each other due to the disruption of eight cascade dams. The population structure of *S. argentatus* was dominated by older and larger individuals, and *S. argentatus* exhibited slightly greater longevity in the middle reaches than in the lower reaches of the Xiangjiang River. The spatial variation in population structure followed a longitudinal gradient along an unregulated river. However, differences in several life‐history traits among the four sampling sections did not align with the common longitudinal river gradients. Specifically, the growth rate was lowest in the midstream sections and lower in the most downstream section. The conditional factor was highest in the uppermost section and lowest in the other three sections. Fecundity was high in the uppermost and lowest sections and low in the two middle sections, whereas egg size showed a decreasing trend along the downstream gradient. According to the egg size/fecundity trade‐off, the longitudinal patterns in fecundity and egg size indicated differences in optimal strategies across the four sampling sections in the middle and lower reaches. The spatial differences in growth rate, conditional factor, and reproductive strategy are apparently related to altered environmental conditions and daily fluctuations in the flow regime of the Xiangjiang River regulated by the eight cascade dams. The dams have disrupted longitudinal gradients in the life‐history traits of *S. argentatus* in the Xiangjiang River.

### 4.1 Longitudinal Patterns of Population Structure and Growth

Growth rate co‐evolves with body size, longevity, and age structure, i.e., fast growth rate generates a smaller body size, shorter lifespan, and a simple age structure, while slower growth rate gives rise to a larger asymptotic length, longer lifespan, and a more complex age structure (Adams et al. 2018; Yang et al. 2026). Fish age and growth patterns, being influenced environmental conditions, vary in space and time (Wakefield et al. 2015; Kornis et al. 2017; Haarr et al. 2018; Huang et al. 2023). Latitudinal gradients in life history traits often follow Bergmann's rule, with populations at higher latitudes showing slower growth, longer longevity, and larger body size (McNicholl et al. 2018; Wright et al. 2020). Patterns of age and growth along a longitudinal river gradient are also well documented (Nakajima and Onikura 2016). It is widely believed that growth rate gradually increases downstream in natural watercourses as elevation and flow velocity decrease and water temperature, habitat dimensions, and nutrient availability increase along the river longitudinal gradient. Along the longitudinal river gradient and the associated increase in growth rate, the asymptotic body size and lifespan decrease, and the age structure is dominated by younger individuals (Tedesco et al. 2009). In this study, the growth rate of *S. argentatus* lacked a clear longitudinal trend along the Xiangjiang River, with high values in the uppermost and third sections and low values in the second heavily regulated section and lowest section immediately below the last cascade dam, These results suggest that cascade dams have disrupted the longitudinal pattern in the growth rate of *S. argentatus* in the Xiangjiang River, as it normally increases downstream. The slower growth rates in the Hangyang and Changsha sections may be owing to environmeantal conditions altered by the cascade dams. Studies have shown that dams can have both positive and negative impacts on fish populations, with effects varying depending on dam characteristics, proximity to the dam, sampling locations, and the specific species and habitats involved. Hydroelectric dams had a minor effect on the growth rates of eight species below E.B. Gampbell Hydroelectric Station on the Saskatchewan River in Canada (Enders et al. 2017; Earley and Sammons 2018). Large dams have been shown to significantly compromise the growth and survival of aquatic species by releasing cold water from the bottom of reservoirs and altering the natural flow regime (Jacquemin et al. 2015; Tonkin et al. 2017; Whiterod et al. 2018). However, dams can promote growth through providing a higher food supply and creating a stable environment in the impoundment reservoir (Kelly et al. 2017). The growth rate in the Hengyang section should have little to do with food availability, because the density and biomass of phytoplankton and zooplankton followed an increasing trend along the downstream gradient. However, the run‐of‐river cascade dams in the middle and lower reaches of the Xiangjiang River have led to sharp diel variation in flow regimes during low‐water seasons and unstable environmental conditions. Specifically, at the Hangyang section, habitats and flow regimes are heavily altered and regulated by the two close‐cascade dams, which are about 50 km apart, and the section has no free‐flowing river habitat. As a result, fish living in unstable environments might spend a large proportion of their energy budget coping with short‐term fluctuations in the flow rates. Furthermore, the deep lentic reservoir has few shallow, slow‐flowing areas beneficial for the growth and survival of the pelagic *S. argentatus* . Another study found that the metabolic rate of brook trout was higher in a regulated river than in a naturally flowing river (Kelly et al. 2017). The lowest section, located immediately downstream of the last cascade dam, experienced a sharp decline in the density and biomass of phytoplankton and zooplankton due to nutrients being intercepted by the dam, and this part of the river has no tributary. Low food availability may be responsible for the slow population growth rate in the lowest reaches. Similarly, Murray cod experienced limited growth directly below the Hume Dam in the Murray‐Darling Basin (Whiterod et al. 2018). Many tributaries entering the Zhuzhou section in the lower reaches can mitigate the effects of upstream cascade dams by adding the runoff volume and transporting nutrients (Katano et al. 2024). Phytoplankton density was relatively low, but zooplankton density was high at the Zhuzhou. Food availability may be not one main reason for the highest growth rate. *Squalidus argentatus* were usually caught in the shallow, slow‐flowing habitats, and the tributaries can offer a large number of such habitats. It may enter into the tributaries for feed and obtain the fast growth rate. A number of studies have found that tributaries provide growth sites and refugia for fish (Da Silva et al. 2019; Moreira et al. 2023). The analysis indicates that the longitudinal variation in the age and growth of *S. argentatum* in the middle and lower reaches of the Xiangjiang River is influenced by the spatial distributions of cascade dams, river reaches, and tributaries.

### 4.2 Trade‐Off Between Fecundity and Egg Size

Female investment in reproduction is determined by environmental conditions experienced during early development and adulthood as well as the growth conditions during spawning (Haslob et al. 2011; Burton et al. 2013). Females in better condition and those with greater energy reserves can allocate more energy to reproduction, thereby producing more offspring. Reproductive investment includes the quantity of spawned eggs and the properties of the oocytes that affect the number and quality of offspring (DöRing et al. 2018; Debes et al. 2025). In this study, the fish population had a significantly higher fecundity and the largest egg size at the uppermost Yongzhou section relative to the other three sites. Evidently, individuals invested more in reproduction at the uppermost site than at the other three sites. The observed high condition coefficient indicates better nutritional conditions and greater energy reserves in the population at the uppermost section, allowing greater investment in reproduction. According to this hypothesis, the population at the uppermost section has experienced benign environmental conditions, with abundant food resources and stable, slow‐flowing habitats at the confluence of the Xiaoshui and Guihe rivers. *Squalidus argentatum* at the Yongzhou section is also the least affected, as it is located upstream of the cascade dams. Similarly, *Pseudobagrus vachellii* exhibited significantly higher fecundity and larger egg size in the lotic zone than in the transitional and lentic zones of the Three Gorges Dam in the Yangtze River. The lotic zone with river habitats had more abundant food resources, whereas the density of zoobenthos is low in the transitional and lentic zones that are affected by the Three Gorges Dam (Liao et al. 2018).

Generally, larger offspring hatch from larger eggs and have a higher survival rate, while a higher fecundity results in more surviving offspring. Offspring size and number are constrained by maternal resources and space, thereby contributing to this trade‐off (Ponce de León et al. 2011; Debes et al. 2025). Consequently, egg number and egg size should be optimized to maximize parental reproductive success and population fitness (Tuor et al. 2020; Debes et al. 2025). The optimal strategy of egg size and fecundity is closely related to environmental conditions, variation in flow regime, and predation pressure, as seen in strategies such as producing a larger number of smaller eggs in resource‐rich environments or fewer, larger eggs in resource‐scarce conditions to maximize the likelihood of offspring survival (Closs et al. 2013; Jones et al. 2016; DöRing et al. 2018). The fecundity/egg size trade‐off is a function of environmental conditions, with fecundity and egg size varying across latitudes, areas, rivers, habitats, and river sections (Haslob et al. 2011; DöRing et al. 2018; Liu et al. 2019). In this study, the increasing pattern in fecundity and the decreasing trend in egg size suggest that low fecundity and large egg size are the optimal reproductive strategies in the middle reaches of the river at Hengyang and Zhuzhou, and fish adopt a strategy of high fecundity and smaller eggs at the Changsha section. *Squalidus argentatus* spawns in the lotic habitats during the high‐water season from April to August, and the eggs drift downstream to slow‐flowing and lentic habitats for development and growth. There were very few optimal nursery habitats at the Hengyang section because the two cascade dams form a deep impounding reservoir. The high water discharge below the dam and the downstream deep impounding reservoir may be harmful for the survival of eggs and development of larvae at the Zhuzhou section. As a result, the inferior nursery grounds and hostile environments force this species to increase egg size for more offspring survival at the Hengyang and Zhuzhou sections. Conversely, population fitness at Changsha was optimized by producing larger numbers of smaller offspring, indicating that the environmental conditions favor the survival and development of eggs and larvae. *Squalidus argentatus* spawns in the lotic habitats below the last cascade dam at the Changsha section, and eggs would drift toward Xiangjiang Estuary, entering Dongting Lake, which represents the most stable and resource‐abundant habitat suitable for the high fecundity and small egg size strategy. Other fish species have shown similar variation in the egg size/fecundity trade‐off along a longitudinal river gradient. *Saurogobio dabryi* had the lowest fecundity and the largest egg size in the middle reaches relative to the upper and lower reaches of a highly regulated tributary (the Jialingjiang River) of the Yangtze River (Liu et al. 2019). The reason may be that cascade dams were constructed in the middle reaches, creating unstable environments and forming deep impounding reservoirs, conditions unfavorable for the survival of small eggs and larvae. In the middle reaches of the Yangtze River, the fecundity of *Pelteobagrus vachellii* and *P. nitidus* was significantly higher in the lotic habitats than in the transitional and lentic habitats close to the Three Gorges Dam. The density of prey (zoobenthos) was higher in the natural lotic habitats of the Yangtze River basin than in other habitats impacted by the dam (Liao et al. 2018). These observations suggest that small/large dams result in a trade‐off characterized by low fecundity and large egg size in regulated rivers/reaches, and natural habitats are crucial for maintaining population productivity.

## 5 Conclusion

In this study, the construction of cascade dams has altered the natural longitudinal growth patterns of fish populations in the Xiangjiang River, leading to varied trade‐offs between egg size and fecundity across different sections in the middle and lower reaches. The population fitness of *S. argentatus* at the Hengyang section was found to be the lowest, potentially due to the significant human impact on the river ecosystem. Growth of the population at this section was hardly affected at the Zhuzhou section in the lower reaches because some natural habitats still exist between the two cascade dams with long distances and many tributaries that could provide growth sites. However, fecundity and egg size were still affected by cascade dams. In the lowest section close to the estuary, the poor prey availability immediately below cascade dams has caused a slow growth rate, but benign environmental conditions in the estuary are suitable for the high fecundity/small egg size strategy. Environmental conditions were least affected by cascade dams and at the confluences of the mainstream and a large tributary, and hence the population exhibited higher growth and the greatest reproductive investment at Yongzhou.

In conclusion, the longitudinal patterns observed in the population structure and growth, as well as the variations in the fecundity/egg size trade‐off along the middle and lower reaches of the Xiangjiang River, suggest that cascade dams with long distance and tributaries can mitigate variation scope of flow regimes and environmental factors and provide feeding grounds. These insights provide theoretical guidance for the construction of cascade dams, emphasizing the need to fully consider distance and location to minimize impacts. The natural habitats upstream of cascade dams and the lowest reaches are crucial for the development and maintenance of fishery resources in the Xiangjiang River, as they are affected by the challenges posed by such developments and the subsequent conservation strategies.

## Author Contributions

**Yanfei Huang:** conceptualization (lead), data curation (lead), formal analysis (lead), funding acquisition (supporting), investigation (lead), methodology (lead), software (lead), supervision (lead), validation (lead), visualization (lead), writing – original draft (lead), writing – review and editing (lead). **Yiyang Gao:** data curation (lead), investigation (lead), methodology (lead), software (lead), validation (equal), visualization (equal). **Yang Zhengfei:** data curation (equal), investigation (lead), methodology (lead), validation (equal), visualization (equal). **Dongzhi Liu:** data curation (equal), methodology (equal), validation (equal), visualization (equal). **LiLi Xie:** data curation (equal), investigation (equal), methodology (equal), validation (equal), visualization (equal). **Deliang Li:** conceptualization (lead), data curation (supporting), formal analysis (equal), funding acquisition (lead), investigation (equal), project administration (lead), resources (lead), supervision (lead), validation (equal), writing – original draft (equal), writing – review and editing (equal).

## Funding

This work was supported by the National Nature Science Foundation of China, 32002394, the Hunan Agriculture Research System, HARS‐7, Research Project of Department of the Agriculture and Rural Affairs of Hunan Province, and the National Key Research and Development Program of China, 2023YFD240092.

## Ethics Statement

The research used fish of *Squalidus argentatus* in the Xiangjiang River as materials. The Chinese Research Ethics Committee has confirmed that no ethical approval is required. The field samplings adhere to a 10‐year ban on Yangtze fishing and were permitted by Department of Agriculture and Rural Affairs of Hunna Province.

## Conflicts of Interest

The authors declare no conflicts of interest.

## Supporting information


**Figure A.1.** The fluctuations of water discharge in a day measured on 23 May, 2025 and 20 August, 2025 and temporal patterns in water discharge throughout a year in 2024 at Yongzhou, Qiyang, and Zhuzhou section of the Xiangjiang River.
**Table A.1**. Environmental variables collected and measured at the four sampling sites of the middle and lower Xiangjiang River.

**Data S1:** The raw data of age, fecundity, egg diameters of Squalidus argentatus collected in the Xiangjiang River.

## Data Availability

All the required data are uploaded as Supporting Information.
