# Supplementary material for: Longitudinal Variation in Population Structure, Growth, and Reproductive Characteristics of Squalidus argentatus, Driven by Cascade Dams in the Largest Tributary of Dongting Lake
Source: Ecol Evol. 2026 Jul 27;16(7):e74081. doi: 10.1002/ece3.74081 (PMC13407322; doi:10.1002/ece3.74081)
Supplement: Supplementary file 1 — Figure A.1. The fluctuations of water discharge in a day measured on 23 May, 2025 and 20 August, 2025 and temporal patterns in water discharge throughout a year in 2024 at Yongzhou, Qiyang, and Zhuzhou section of the Xiangjiang River. Table A.1. Environmental variables collected and measured at the four sampling sites of the middle and lower Xiangjiang River. [file ECE3-16-e74081-s001.docx]

**Table A.1** Environmental variables collected and measured at the four sampling sites of the middle and lower Xiangjiang River.

| Date | Yongzhou | Hengyang | Zhuzhou Dam | | Changsha Dam | |
| --- | --- | --- | --- | --- | --- | --- |
|  |  |  | Above | Below | Above | Below |
| Phytoplankton density (cells/L) | | | | | | |
| Jun-24 | 389527 | 777712 | 999273 | 219066 | 284083 | 252975 |
| Oct-24 | 162500 | 458333 | – | 270833 | 375000 | 281250 |
| Zooplankton density (ind/L) | | | | | | |
| Jun-24 | 88.4 | 140.1 | ‒ | 190.3 | 550.3 | 93.1 |
| Jul-25 | 60.0 | 92.1 | 225.9 | 91.2 |  |  |
| Velocity (m/s) | | | | | | |
| May-23 | 0.50 | 0.10 | 0.0 | 0.30 | 0.10 | 0.20 |
| Jul-25 | 0.25 | 0.12 | 0.0 | 0.24 | 0.13 | 0.26 |
| River width (Mean ± SD, m) | | | | | | |
| Jun-25 | 341 ± 67 | 472 ± 154 | 645 ± 64 | 642 ± 126 | 922 ± 124 | 950 ± 79 |

Fig. A.1 The fluctuations of water discharge in a day measured on 23 May, 2025 and 20 August, 2025 and temporal patterns in water discharge throughout a year in 2024 at Yongzhou, Qiyang, and Zhuzhou section of the Xiangjiang River.
